# Isolated Apophyseal Avulsion of the Greater Trochanter Treated by Minimally Invasive Osteosynthesis: A Case Report

**DOI:** 10.5704/MOJ.2011.029

**Published:** 2020-11

**Authors:** G Malerba, M Basilico, N Bonfiglio, G Maccauro

**Affiliations:** Department of Orthopaedics and Traumatology, Fondazione Policlinico Universitario A. Gemelli IRCCS, Rome, Italy

**Keywords:** greater trochanter, apophysis avulsion, osteonecrosis

## Abstract

Isolated apophyseal avulsion of the greater trochanter is a rare condition in the paediatric population, frequently related to avascular necrosis of the femoral head. Since there are few cases in the literature, there is no consensus regarding the best treatment of this injury. Our study describes the case of a 9-year-old patient with an avulsion of the right greater trochanter. A minimally invasive osteosynthesis was performed, achieving complete clinical and radiographic healing of the patient and no long-term complications after four years.

## Introduction

Isolated avulsion of the greater trochanter apophysis is a rare condition in the paediatric population and is generally due to direct trauma or a sudden contraction against resistance in the gluteus muscles. In the published literature, this injury has been reported in very few cases and it is frequently associated with avascular necrosis (AVN) of the femoral head^1-2^. Other complications are not reported in the literature. Its management is controversial and it is not clear whether a non-operative or surgical treatment should be recommended.

The aim of this study is to describe the case of a 9-year-old patient with an avulsion of the right greater trochanter treated with single screw fixation. The patient and his family were informed that the case data would be submitted for publication and provided consent.

## Case Report

A 9-year-old female patient presented to our Emergency Department with pain in the right hip after high-energy direct trauma as a result of high-speed motor vehicle accident. On examination, hematoma and swelling were present in the lateral region of the thigh, the right hip was in external rotation and slight flexion and the knee flexed. The hip pain worsened on palpation of the greater trochanter region and prevented weight-bearing and movement of hip and knee. Circulation and neurological examination were normal, the skin was intact.

**Fig. 1:**
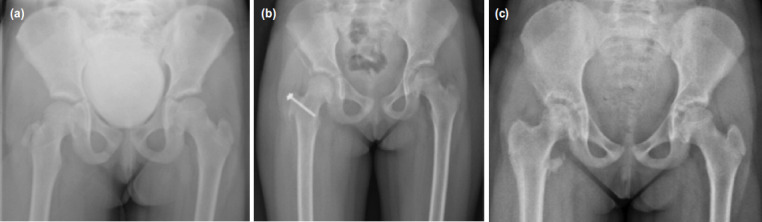
(a) Anteroposterior radiographs of the right hip at the time of presentation, (b) after surgery (c) and after screw removal.

**Fig. 2:**
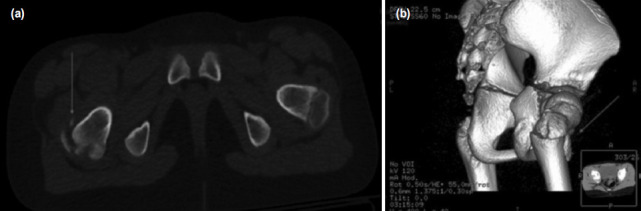
(a) Axial view (b) and 3D reconstruction CT imaging of the pelvis, made at the time of presentation, showing three-fragment fracture of the greater trochanter with the biggest fragment displaced anteriorly (marked by the arrow).

Pelvis radiographs showed an avulsion of the right greater trochanter (Fig. 1a) and computed tomography (CT) imaging revealed a three-fragment fracture of the greater trochanter with the biggest fragment displaced anteriorly, indicative of injury following direct trauma to the hip (Fig. 2). Considering the significant displacement of the greater trochanter, surgical treatment was preferred.

The following day the patient underwent a minimally invasive osteosynthesis. A 2cm peritrochanteric incision was made on the lateral side of the greater trochanter. After bluntly splitting the gluteus maximus, the displaced trochanter was reduced with femoral internal rotation, avoiding any direct manipulation of the fragments. Guided by image intensifier, the fixation was performed through a single 4.5mm cannulated screw with washer (Fig. 1b), taking care not to tighten the screw tightly in order to reduce the risk of iatrogenic growth plate injury. No further screws were needed, as the reduction was stable enough to ensure retensioning of the external rotators. The patient was mobilised immediately after the surgery with protected weight-bearing and crutches, no braces were used. Full weight-bearing was allowed six weeks after surgery. Two months after surgery clinical examination revealed pain-free hip movement and deambulation and a hip radiograph demonstrated a stable alignment of the fragments. At six months, the patient was clinically and radiographically healed, so screw removal was performed (Fig. 1c).

**Fig. 3:**
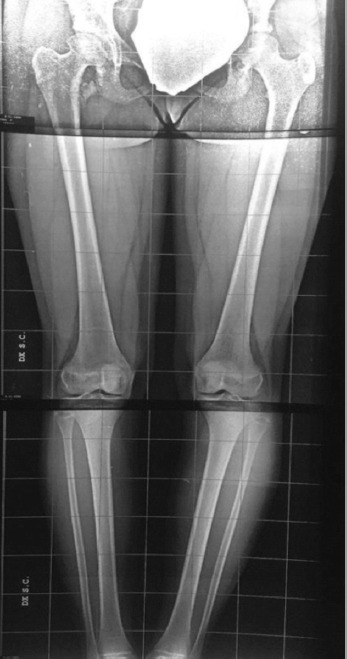
Underloading lower limb radiograph, made four years after the injury, demonstrating total healing of the greater trochanter, a dysmorphic right femoral head and no leg length discrepancy.

During the latest visit, four years after the injury, the scanogram showed no length discrepancy but, by comparing both sides, the right femoral head appears dysmorphic. Since the patient had full pain-free deambulation and sport activity, no further investigation was required, but only a closer follow-up (Fig. 3).

## Discussion

An analysis of the literature reveals that avulsion fractures of the greater trochanter are a rare injury among the paediatric population, but it may frequently lead to subsequent development of avascular necrosis of the femoral head^1-5^. This serious complication is possible in young patients: whilst the physis is open the lateral ascending cervical artery and the medial circumflex femoral artery provide vascular supply to the femoral head^1^. AVN of the femoral head after greater trochanter apophyseal avulsion could occur due to a lesion of these vessels: the causes include vessels traction or injury as a direct consequence of the trauma or indirect of the intracapsular hematoma or iatrogenic vascular damage at the time of the surgery^1-2^.

Since few cases are reported in the literature, recommendations are not unanimous regarding the best strategy between conservative and operative management programs (open or closed reduction and internal fixation). Poor outcomes have been reported following both treatments, but surgery seems to be the most popular treatment. Wood *et al* described a successful clinical and radiographic outcome after open reduction and internal fixation of an avulsion fracture of the greater trochanter in a 15-year-old boy^3^. The same positive outcome is reported by El Hachmi *et al* after percutaneous treatment in a 13-year-old boy^4^.

Very few authors focused on conservative treatment for this condition. Giles reported excellent treatment outcome in a 14 years old boy with a minimally displaced greater trochanter avulsion fracture managed with a hip abduction brace and limit weight-bearing^5^. Finally, O’Rourke and Weinstein described two cases of AVN both after conservative and surgical management, in a 5-year-old and a 13-year-old patient, respectively^2^, whereas Macdonald *et al* presented a 14-year-old patient with greater trochanter avulsion successfully managed non-operatively^1^.

In conclusion, high risk of avascular necrosis of the head of the femur should be considered in planning the management of avulsion of the greater trochanter apophysis. Available evidence and our own experience support minimally invasive surgical treatment. An indirect reduction, without direct manipulation of the fragment, and an osteosynthesis using a single cannulated screw, avoiding affecting the bands tendon and soft tissues, minimise vascular risk. Conservative treatment might be limited where the diastasis of the apophysis of the greater trochanter is minimal.
